# Ameliorative effects of zinc supplementation on cognitive function and hippocampal leptin signaling pathway in obese male and female rats

**DOI:** 10.1038/s41598-023-31781-8

**Published:** 2023-03-28

**Authors:** Lamia M. Hafez, Hebatallah Mohammed Aboudeya, Noura A. Matar, Ashraf S. El-Sebeay, Azhar Mohamed Nomair, Shaymaa Ali El-hamshary, Hanan Mohamed Nomeir, Fawziya A. R. Ibrahim

**Affiliations:** 1 Human Nutrition Department, Regional Center for Food and Feed-Agricultural Research Center, Alexandria, Egypt; 2 Human Physiology Department, Medical Research Institute, Alexandria University, Alexandria, Egypt; 3 Department of Histochemistry and Cell Biology Medical Research Institute, Alexandria University, Alexandria, Egypt; 4 Department of Chemical Pathology, Medical Research Institute, Alexandria University, Alexandria, Egypt; 5 Biochemistry Department, Faculty of Science, Alexandria University, Alexandria, Egypt; 6 Department of Medical Biochemistry, Faculty of Medicine, Alexandria University, Alexandria, Egypt; 7 Applied Medical Chemistry Department, Medical Research Institute, Alexandria University, 165, Horreya Avenue, Hadara, Alexandria, Egypt

**Keywords:** Biochemistry, Pathogenesis

## Abstract

Obesity has been associated with cognitive impairments, increasing the probability of developing dementia. Recently, zinc (Zn) supplementation has attracted an increasing attention as a therapeutic agent for cognitive disorders. Here, we investigated the potential effects of low and high doses of Zn supplementation on cognitive biomarkers and leptin signaling pathway in the hippocampus of high fat diet (HFD)-fed rats. We also explored the impact of sex difference on the response to treatment. Our results revealed a significant increase in body weight, glucose, triglycerides (TG), total cholesterol (TC), total lipids and leptin levels in obese rats as compared to controls. HFD feeding also reduced brain-derived neurotrophic factor (BDNF) levels and increased acetylcholinesterase (AChE) activity in the hippocampus of both sexes. The low and high doses of Zn supplementation improved glucose, TG, leptin, BDNF levels and AChE activity in both male and female obese rats compared to untreated ones. Additionally, downregulated expression of leptin receptor (LepR) gene and increased levels of activated signal transducer and activator of transcription 3 (p-STAT3) that observed in hippocampal tissues of obese rats were successfully normalized by both doses of Zn. In this study, the male rats were more vulnerable to HFD-induced weight gain, most of the metabolic alterations and cognition deficits than females, whereas the female obese rats were more responsive to Zn treatment. In conclusion, we suggest that Zn treatment may be effective in ameliorating obesity-related metabolic dysfunction, central leptin resistance and cognitive deficits. In addition, our findings provide evidence that males and females might differ in their response to Zn treatment.

## Introduction

Obesity is one of the most important health issues of the twenty-first century, and its prevalence has increased at an alarming rate. According to recent estimates, 38% of men and 40% of women (1.9 billion) are overweight, with 11% and 14% (600 million) being obese^1^. More recently, obesity has been identified as a substantial risk factor for mild cognitive impairment, dementia, and Alzheimer's disease^2^. Through leptin receptors (LepRs) expressed on the hypothalamus nuclei, the adipokine leptin can regulate food intake, metabolism, and body weight^2,3^. LepRs have been identified in the hippocampus, and emerging evidence suggests that leptin promotes synaptic plasticity and improves learning and memory performance, whereas central leptin resistance is associated with memory loss^4^. LepRs downregulation and/or inadequate leptin signaling downstream of LepRs are linked to central leptin resistance^5^.

Zinc (Zn) is one of the most abundant trace elements required for brain activities and its deficiency is associated with neuronal degeneration and cognitive impairment^6^. It is highly concentrated in the amygdale, the auditory brain stem, the cerebral cortex, and the hippocampus^7,8^. Zn functions in the brain as a neurotransmitter and second messenger, controlling hippocampus long-term potentiation, boosting neuronal survival, and promoting learning and memory^6^.

Zn deficiency, on the other hand, is linked to obesity, insulin resistance, and hyperglycemia^9–11^. In obese animals, Zn supplementation can alleviate obesity, enhance insulin sensitivity, and reduce leptin levels^12^. However, Zn’s effect on cognitive function in obese condition and underlying mechanisms are still unknown. Furthermore, although a sex-related link between obesity and cognitive impairment has been investigated in various researches^13–16^, the impact of sex difference on response to Zn treatment effects needs more studies to be established.

In this context, the present study aimed to investigate the effects of different doses (low and high doses) of Zn supplementation on cognitive biomarkers and leptin signaling pathway in the hippocampus of high fat diet (HFD)˗fed rats and to determine sex difference impact on response to Zn supplementation.

## Materials and methods

### Ethical statement

All animal procedures and experimental protocols were performed in accordance with the International Guiding Principles for Biomedical Research Involving Animals^17^ and were approved by Institutional Animal Care and Use Committee (IACAUC), Medical Research Institute of Alexandria University. The current study adheres to the ARRIVE Guidelines for reporting in vivo experiments^18^.

### Animals and experimental design

This study was conducted on 72 albino Sprague–Dawley rats (36 males and 36 females) (6 weeks old, 165 g average weight), purchased from the animal house of Institute of Graduate Studies and Research, Alexandria, Egypt. All rats were housed under the following condition; 12 h dark/light cycle at room temperature and humidity between 30 and 70% access to water, animals were divided in individual plastic cages 6 rats per cage with soft bedding.

Male as well as female rats were divided into two main groups (18 in each group): control diet (CD) and high fat diet (HFD) fed groups, (HFD) contains 45% Kcal from fats (D12451; Research Diets, Inc., New Brunswick, NJ, USA)^19^.

Progression of obesity was monitored through determination of the increase in body weight, serum levels of total lipids, cholesterol, and triglycerides in CD and HFD-fed rats, Table (1). After 8 weeks, rats were further divided into total six groups (6 rats each); CD, CD-low Zn (15 mg/kg ZnSO_4_.7H_2_O); Sigma, St. Louis, MO), CD-high Zn (30 mg/kg ZnSO_4_.7H_2_O), HFD, HFD-low Zn and HFD-high Zn groups. Zn solution was daily administered using oral gavage for 4 weeks after its preparation by dissolving water-soluble zinc sulfate (ZnSO_4_.7H_2_O) in distilled water^20^.

**Table 1:** Body weight, serum glucose level, and lipid profile in control versus HFD groups after 8 weeks in males and females rats.

Groups	Control		HFD	
	Males	Females	Males	Females
Weight (g)	228.2 ± 37.7^a^	188.9 ± 8.0^b^	306.3 ± 36.4^a^	230.1 ± 8.8^b^
Glucose (mg/dl)	95.0 ± 8.0^b^	96.0 ± 10.2^b^	127.9 ± 8.3^a^	128.1 ± 8.1^a^
Total lipids (mg/dl)	301.8 ± 44.2^b^	220.7 ± 4.7^b^	367.7 ± 17.0^a^	278.8 ± 8.2^a^
Triglycerides (mg/dl)	91.8 ± 15.2^b^	159.1 ± 34.9^a^	155.1 ± 37.6^a^	188.7 ± 66.0^a^
Cholesterol (mg/dl)	79.4 ± 7.1^b^	92.1 ± 10.5^a^	103.1 ± 14.9^a^	1174.2 ± 37.2^a^

Data was expressed using mean ± SD.

Means in the same raw with small common letters are not significant (i.e. means with different letters are significant).

*SD* standard deviation.

At the end of the experiment, all rats were fasted for 12 h, blood and brain tissues were collected after animals exposed to deep anesthesia using isoflurane, then all animals were terminated by decapitation.

### Sample collection

A blood sample was collected from each rat, allowed to clot at room temperature and centrifuged at 3000 rpm; the aspirated serum was stored at − 20 °C till used for assay of glucose, leptin, Zn concentrations, lipid profile, liver and kidney functions. Brain was dissected immediately to isolate the hippocampus. The first part of hippocampal tissues of studied groups was homogenized, centrifuged and supernatants were used for determination of brain-derived neurotrophic factor (BDNF), acetylcholine esterase (AChE) and phospho-STAT3 (p-STAT3) levels. The second part was used for RNA extraction to measure the gene expression of leptin receptor (LepR). For histopathological studies, the third portion was preserved in 10% formalin.

### Biochemical serum analysis

Serum aspartate aminotransferase (AST/GOT) and alanine aminotransferase (ALT/GPT), total lipids, triglycerides (TG), total cholesterol (TC), fasting glucose levels, urea, uric acid, and creatinine were assayed using commercial kits (Biosystems, Costa Brava 30, 08030 Barcelona, Spain).

Serum leptin levels were quantified using commercially available sandwich ELISA kit purchased from (BIOMATIK, 9-14 McGovern. Drive, Cambridge, Ontario, N3H4R7, Canada), according to the manufacturer’s instructions. The concentration of Zn (µg/dl) was assayed by atomic absorption spectroscopy (AAS) (SPECTRA AA-PLUS VERSION, working with air/acetylene flame and D2-background correction)^21^.

### Determination of AChE activity, BDNF, and p-STAT3 in hippocampal tissues

Hippocampal tissue samples were homogenized in 0.1 M phosphate buffer, PH 7.5, followed by centrifugation at 14,000 rpm for 5 min, and then the supernatant was used for determination of AChE activity using AChE activity assay kit (MAK 119, Sigma Alderich). In addition, BDNF and p-STAT3 concentrations were assayed in hippocampus using sandwich ELISA kits purchased from (MyBioSource, Inc., San Diego, CA, USA and Thermo Fisher Scientific, Waltham, MA, USA), respectively.

### Quantitative reverse transcriptase polymerase chain reaction (q-RT-PCR) of LepR gene expression in hippocampal tissues

Total RNA was extracted from brain tissues using Easy-RED Total RNA Extraction Kit (iNtRON Biotechnology Inc., South Korea) according to the manufacturer’s instructions. The purity and concentration of extracted RNA were evaluated by NanoDrop ND-1000 UV–Visible Spectrophotometer (Thermo Fischer Scientific, USA). Total RNA was reverse transcribed using TOPscript RT DryMixsynthesis kit (Enzynomics Inc., South Korea), following the manufacturer’s instructions. The PCR reaction was carried in 20 μl final volume, where 2 μl cDNA, 1.25 μl of the each forward and reverse primer (10pMol/µl), 10 μl (TOPreal ™ qPCR 2X PreMIX) SYBER Green master mix, and 5.5 μl RNAse-free water were dispensed into the PCR tubes. The amplification profile was as follow, initial denaturation at 95 for 15 min, followed by 35 cycles of denaturation at 95 for 10 s, annealing at 60 for 15 s, and final elongation at 72 for 30 s. The relative expression of leptin receptor was quantified relative to the expression of 18S ribosomal RNA (18S rRNA) as a reference gene, 2^−∆∆ct^ equation was used to calculate LepR gene expression. Primer sequence for leptin receptor and 18S rRNA are represented as follows.

**Table:** 

Gene	Primer sequence	
Leptin receptor	F: CTGGCTGTCTCACATCTCCC	R: AATGAGCGTTCTTCCAACCCCC
18S rRNA	F: GTAACCCGTTGAACCCATT	R: CAAGCTTATGACCCGCACTT

### Histopathological studies

The hippocampal samples were fixed with 10% neutral buffered formalin, and then passed through ascending concentrations of ethanol, cleared in xylene and embedded in paraffin. Using a rotary microtome, paraffin sections of 4 μm thickness were cut and stained with hematoxylin and eosin (H&E). Under light microscope, the hippocampus was examined for any histopathological changes.

### Statistical analysis

Data were fed to the computer and analyzed using IBM SPSS software package version 20.0***. (***Armonk, NY: IBM Corp). The normality of the distribution was confirmed using the Kolmogorov–Smirnov test, and parametric data were represented using mean ± standard deviation. Two way ANOVA test was used to analyze the effect of zinc treatment, gender, and the possible interaction between zinc treatment and gender variation on all the studied parameters, Table (2). One way ANOVA was used to compare between more than two groups, and Post Hoc test (Tukey) for pairwise comparisons. p value ≤ 0.05 was considered statistically significant.

**Table 2:** Two way ANOVA analyses of all studied parameters in all groups regarding gender, Zn treatment, and gender versus ZnSO_4_ treatment.

Studied parameters	Zn-treatment		Gender		Zn-treatment/gender	
	F	p	F	P	F	p
Total weight	118.65	˂ 0.0001	746.18	˂ 0.0001	5.41	0.0002
Serum markers						
Zn	53.72	˂ 0.0001	380.63	˂ 0.0001	11.51	˂ 0.0001
Leptin	228.51	˂ 0.0001	2045.89	˂ 0.0001	45.45	˂ 0.0001
Glucose level	80.56	˂ 0.0001	0.00	0.9756	3.74	0.0034
Brain markers						
Leptin receptor	228.51	˂ 0.0001	2045.89	˂ 0.0001	45.45	˂ 0.0001
PDNF	1246.42	˂ 0.0001	465.32	˂ 0.0001	14.46	˂ 0.0001
p-STAT3	283.27	˂ 0.0001	155.63	˂ 0.0001	4.87	0.0004
AChE	6.63	˂ 0.0001	61.75	˂ 0.0001	33.81	˂ 0.0001
Lipid profile						
Total Lipids	23.4	˂ 0.0001	365.91	˂ 0.0001	2.91	0.0158
Cholesterol	20.18	˂ 0.0001	147.03	˂ 0.0001	1.21	0.3072
Triglycerides	110.47	˂ 0.0001	128.92	˂ 0.0001	15.24	˂ 0.0001
Liver enzymes						
SGOT	34.78	˂ 0.0001	150.27	˂ 0.0001	15.22	˂ 0.0001
SGPT	134.15	˂ 0.0001	445.15	˂ 0.0001	37.49	˂ 0.0001
Kidney functions test						
Urea	16.3	˂ 0.0001	4.54	0.0350	0.51	0.7681
Uric acid	39.25	˂ 0.0001	9.38	0.0027	11.90	˂ 0.0001
Creatinine	2.02	0.0798	7.83	0.0059	1.55	0.1791

## Results

### Effect of Zn treatment on anthropometric and biochemical parameters

As shown in Tables (3, 4), body weight and serum levels of glucose, total lipids, cholesterol and triglycerides were significantly increased in obese male and female rats as compared to controls (p ≤ 0.05).

**Table 3:** Body weight, serum glucose level, lipid profile, liver and kidney functions in control and obese groups without ZnSO_4_ tretatment, low dose (15 mg) ZnSO_4_ treatment, and high dose (30 mg) ZnSO_4_ treatment in male rats.

Studied parameters	Control group			Obese group		
	Without ZnSO_4_	Low dose ZnSO_4_	High dose ZnSO_4_	Without ZnSO_4_	Low dose ZnSO_4_	High dose ZnSO_4_
Body weight (g)	313.4 ± 22.8^bc^	300.6 ± 6.4^bc^	289.4 ± 24.9^c^	419.0 ± 8.2^a^	324.3 ± 42.7^b^	303.9 ± 20.4^b^
Glucose (mg/dl)	92.7 ± 10.7^cd^	93.7 ± 7.7^bcd^	91.0 ± 6.6^cd^	130.3 ± 3.5^a^	94.2 ± 6.6^bcd^	96.7 ± 9.8^bc^
Lipid profile						
Lipids (mg/dl)	347.4 ± 19.1^b^	347.9 ± 21.8^b^	323.5 ± 25.4^bc^	406.5 ± 22.6^a^	355.7 ± 14.4^b^	343.3 ± 16..2^bc^
Cholesterol (mg/dl)	97.0 ± 7.3^bc^	97.7 ± 14.2^bc^	99.0 ± 26.8^bc^	129.0 ± 10.3^a^	111.0 ± 28.0^ab^	112.3 ± 13.6^ab^
Triglyceride (mg/dl)	94.7 ± 10.4^c^	90.0 ± 18.2^c^	90.7 ± 15.0^c^	196.3 ± 17.3^a^	157.3 ± 7.4^b^	153.3 ± 12.0^b^
Liver enzymes						
GOT (U/L)	167.3 ± 13.2^bcd^	162.2 ± 13.6^cde^	131.4 ± 13.0^ef^	275.0 ± 57.1^a^	199.2 ± 27.7^b^	173.0 ± 10.7^bc^
GPT (U/L)	63.1 ± 4.1^b^	48.9 ± 7.6^cd^	47.0 ± 8.9^cde^	93.1 ± 5.0^a^	40.9 ± 5.4^ef^	41.1 ± 5.8^def^
Kidney functions						
Urea (mg/dl)	34.0 ± 2.0^e^	37.3 ± 3.0^bcde^	37.0 ± 4.3^cde^	42.2 ± 2.6^a^	38.3 ± 1.9^abcd^	38.4 ± 2.1^abc^
Uric acid (mg/dl)	2.6 ± 0.4^e^	3.0 ± 0.4^de^	3.3 ± 0.5^cde^	2.6 ± 0.4^e^	3.3 ± 0.6^cde^	3.4 ± 0.2^cd^
Creatinine (mg/dl)	0.6 ± 0.07^ab^	0.7 ± 0.07^a^	0.6 ± 0.06^ab^	0.6 ± 0.05^ab^	0.6 ± 0.07^ab^	0.6 ± 0.08^ab^

Data was expressed using mean ± SD.

Means in the same raw with small common letters are not significant (i.e. means with different letters are significant).

*SD* standard deviation.

**Table 4:** Body weight, serum glucose level, lipid profile, liver and kidney functions in control and obese groups without ZnSO_4_ tretatment, low dose (15 mg) ZnSO_4_ treatment, and high dose (30 mg) ZnSO_4_ treatment in female rats.

Studied parameters	Control group			Obese group		
	Without ZnSO_4_	Low dose ZnSO_4_	High dose ZnSO_4_	Without ZnSO_4_	Low dose ZnSO_4_	High dose ZnSO_4_
Body weight (g)	215.6 ± 11.4^d^	209.8 ± 14.8^d^	205.6 ± 8.8^de^	331.4 ± 16.3^b^	189.1 ± 7.2^de^	178.3 ± 30.5^e^
Glucose (mg/dl)	83.0 ± 14.5^d^	89.6 ± 3.0^cd^	93.7 ± 9.0^cd^	132.2 ± 11.0^a^	105.0 ± 9.8^b^	95.8 ± 5.2^bc^
Lipid profile						
Lipids (mg/dl)	256.0 ± 11.8^ef^	232.4 ± 17.7^f^	237.4 ± 29.2^f^	307.2 ± 18.4^cd^	283.2 ± 36.3^de^	281.7 ± 40.5^de^
Cholesterol (mg/dl)	69.1 ± 12.1^de^	58.3 ± 8.8^e^	54.3 ± 10.3^e^	102.6 ± 8.4^bc^	81.2 ± 9.1^cd^	82.7 ± 25.6^cd^
Triglyceride (mg/dl)	149.0 ± 17.5^b^	146.7 ± 5.1^b^	141.2 ± 23.0^b^	218.7 ± 27.8^a^	153.7 ± 20.3^b^	160.2 ± 6.9^b^
Liver enzymes						
GOT (U/L)	142.8 ± 23.4^cdef^	111.0 ± 25.0^f^	128.4 ± 29.0^ef^	154.8 ± 15.8^cde^	133.1 ± 15.6^def^	128.8 ± 21.2^ef^
GPT (U/L)	31.8 ± 4.0^gh^	27.6 ± 4.6^ h^	30.2 ± 5.3^gh^	51.6 ± 7.9^c^	35.92 ± 4.4^ fg^	35.6 ± 3.3^ fg^
Kidney functions						
Urea (mg/dl)	34.4 ± 1.3^de^	35.7 ± 3^.^0^cde^	36.4 ± 2.3^cde^	41.1 ± 4.5^a^	36.2 ± 3.6^cde^	36.9 ± 3.1^cde^
Uric acid (mg/dl)	3.6 ± 0.3^cd^	3.7 ± 0.3^cd^	3.8 ± 0.3^bc^	4.5 ± 1.3bs	3.7 ± 0.4^cd^	3.7 ± 0.5^cd^
Creatinine (mg/dl)	0.6 ± 0.02^ab^	0.6 ± 0.09^ab^	0.6 ± 0.08^ab^	0.5 ± 0.08^b^	0.6 ± 0.08^ab^	0.5 ± 0.07^b^

Data was expressed using Mean ± SD.

Means in the same raw with small common letters are not significant (i.e. means with different letters are significant).

*SD* standard deviation.

Low and high Zn doses significantly decreased body weight as well as serum levels of glucose and triglycerides in both obese male and female rats compared to untreated obese rats (p ≤ 0.05). A significant decrease in serum levels of total lipids was also detected in obese male rats (p ≤ 0.05). In contrast, the treatment with either doses didn’t show any significant effect on cholesterol levels in male and female obese rats compared to untreated obese rats, Tables (3, 4).

Regarding sex variations, obese males showed a significant increase in body weight and serum levels of total lipids and cholesterol compared to obese females (p ≤ 0.05). However, no significant differences were detected in serum levels of glucose and triglycerides. After treatment, obese females showed a significant decrease in total lipids and TC levels compared to obese males, Table (5).

**Table 5:** Comparison between body weight, serum glucose level, lipid profile, liver and kidney functions in males versus female obese groups without ZnSO_4_ tretatment, low dose (15 mg) ZnSO_4_ treatment, and high dose (30 mg) ZnSO_4_ treatment.

Studied parameters	Male obese group			Female obese group		
	Without ZnSO_4_	Low dose ZnSO_4_	High dose ZnSO_4_	Without ZnSO_4_	Low dose ZnSO_4_	High dose ZnSO_4_
Body weight (g)	419 ± 8.2^a^	324.3 ± 42.7	303.9 ± 20.4	331.4 ± 16.3^b^	189.1 ± 7.2^de^	178.3 ± 30.5^e^
Glucose (mg/dl)	130.3 ± 3.5^a^	94.2 ± 6.6^bcd^	96.7 ± 9.8^bc^	132.2 ± 11.0^a^	105.0 ± 9.8^b^	95.8 ± 5.2^bc^
Lipid profile						
Lipids (mg/dl)	406.5 ± 22.6^a^	355.7 ± 14.4^b^	343.3 ± 16..2^bc^	307.2 ± 18.4^cd^	283.2 ± 36.3^de^	281.7 ± 40.5^de^
Cholesterol (mg/dl)	129.0 ± 10.3^a^	111.0 ± 28.0^ab^	112.3 ± 13.6^ab^	102.6 ± 8.4^bc^	81.2 ± 9.1^cd^	82.7 ± 25.6^cd^
Triglyceride (mg/dl)	196.3 ± 17.3^a^	157.3 ± 7.4^b^	153.3 ± 12.0^b^	218.7 ± 27.8^a^	153.7 ± 20.3^b^	160.2 ± 6.9^b^
Liver enzymes						
GOT (U/L)	275.0 ± 57.1^a^	199.2 ± 27.7^b^	173.0 ± 10.7^bc^	154.8 ± 15.8^cde^	133.1 ± 15.6^def^	128.8 ± 21.2^ef^
GPT (U/L)	93.1 ± 5.0^a^	40.9 ± 5.4^ef^	41.1 ± 5.8^def^	51.6 ± 7.9^c^	35.92 ± 4.4^ fg^	35.6 ± 3.3^ fg^
Kidney functions						
Urea (mg/dl)	42.2 ± 2.6^a^	38.3 ± 1.9^abcd^	38.4 ± 2.1^abc^	41.1 ± 4.5^a^	36.2 ± 3.6^cde^	36.9 ± 3.1^cde^
Uric acid (mg/dl)	2.6 ± 0.4^e^	3.3 ± 0.6^cde^	3.4 ± 0.2^cd^	4.5 ± 1.3^bs^	3.7 ± 0.4^cd^	3.7 ± 0.5^cd^
Creatinine (mg/dl)	0.6 ± 0.05^ab^	0.6 ± 0.07^ab^	0.6 ± 0.08^ab^	0.5 ± 0.08^b^	0.6 ± 0.08^ab^	0.5 ± 0.07^b^

Data was expressed using mean ± SD.

Means in the same raw with small common letters are not significant (i.e. means with different letters are significant).

*SD* Standard deviation.

### Effect of Zn treatment on liver and kidney function tests

The serum levels of GPT and urea were significantly increased in obese rats of both sexes compared to their corresponding controls (p ≤ 0.05), whereas uric acid levels were significantly increased only in obese female rats (p ≤ 0.05), Tables (3, 4).

In obese males, low and high doses of Zn significantly lowered serum GOT and GPT levels (p ≤ 0.05). In obese females, both doses significantly decreased GPT, urea and uric acid serum levels (p ≤ 0.05) compared to untreated obese rats, Tables (3, 4).

As compared to obese females, obese males showed a significant elevation in the levels of GOT, GPT (p ≤ 0.05). After Zn treatment, obese females showed a significant reduction in serum GOT as compared to obese males, Table (5).

### Effect of zinc treatment on leptin and zinc levels

**Figure 1:**
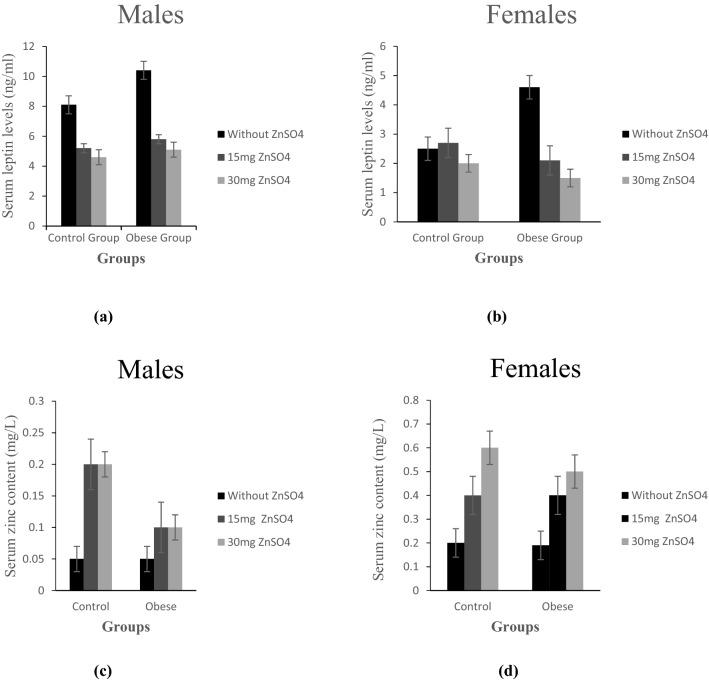
(**a**,**b**) Serum levels of leptin (ng/ml) in control and obese groups; without ZnSO_4_, 15 mg ZnSO_4_, and 30 mg ZnSO_4_; in male rats and female rats, respectively; (**c**,**d**) zinc content in control and obese groups; without ZnSO_4_, 15 mg ZnSO_4_, and 30 mg ZnSO_4_; in male and female rats, respectively; Results are expressed as (mean ± S.D).

The results in Fig. (1a,b) indicated a significant increase in serum leptin in male and female obese rats, which was reversed by both doses of supplemented Zn (p ≤ 0.05).

Regarding serum Zn levels, there wasn’t any significant difference in either obese male or female groups as compared to control. Treatment with low and high Zn doses increased Zn levels in both male and female obese rats as compared to untreated rats, however, this increase was significant in obese female rats only (p ≤ 0.05), Fig. (1c,d).

As compared to obese females, obese males showed a significant decrease in Zn levels and a significant increase in leptin levels (p ≤ 0.05). After Zn treatment, their levels were reversed significantly in obese females compared to obese males, Fig. (4a,b).

### Effect of zinc treatment on cognitive biomarkers (hippocampal AChE activity and PDNF levels)

**Figure 2:**
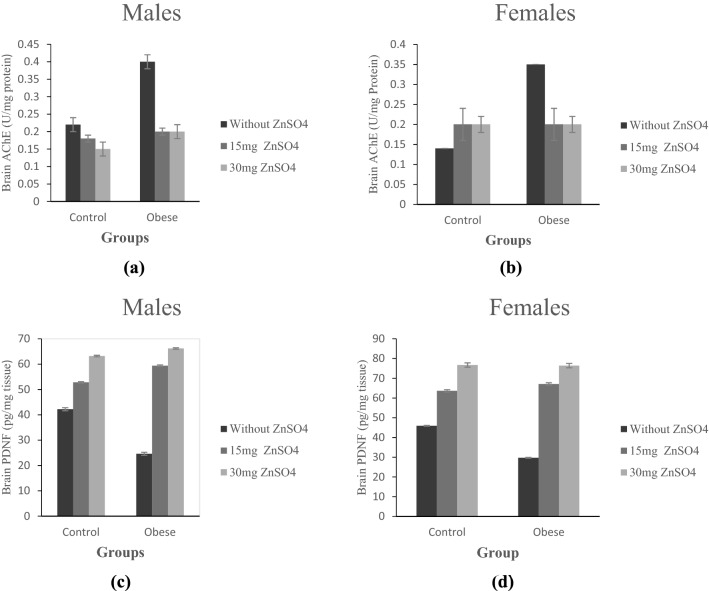
(**a**,**b**) Brain tissue levels of AChE (U/mg tissue) in control and obese groups; without ZnSO_4_, 15 mg ZnSO_4_, and 30 mg ZnSO_4_ in male and female rats, respectively; (**c**,**d**) Brain levels of PDNF (pg/mg tissue) in control and obese groups; without ZnSO_4_, 15 mg ZnSO_4_, and 30 mg ZnSO_4_; in in male and female rats, respectively. Results are expressed as (mean ± S.D).

The present results indicated a significant increase in hippocampal AChE activity and a significant decrease in PDNF levels in obese male and female rats as compared to their control counterparts (p ≤ 0.05), Fig. (2a–d).

Treatment with either low or high Zn doses significantly decreased AChE activity in obese male and female rats compared to untreated obese rats (p ≤ 0.05), Fig. (2a,b). On the other side, hippocampal PDNF levels were significantly increased in both sexes of obese treated groups compared to untreated groups (p ≤ 0.05), Fig. (2c,d).

As compared to obese females, obese male rats showed a significantly higher levels of AChE activity and significantly lower PDNF levels (p ≤ 0.05), Fig. (4c,d). After treatment, AChE activity were significantly decreased, while PDNF levels were significantly increased in obese females compared to obese males.

### Effect of zinc treatment on leptin signaling in the hippocampus (leptin receptor gene expression and p-STAT3 levels)

**Figure 3:**
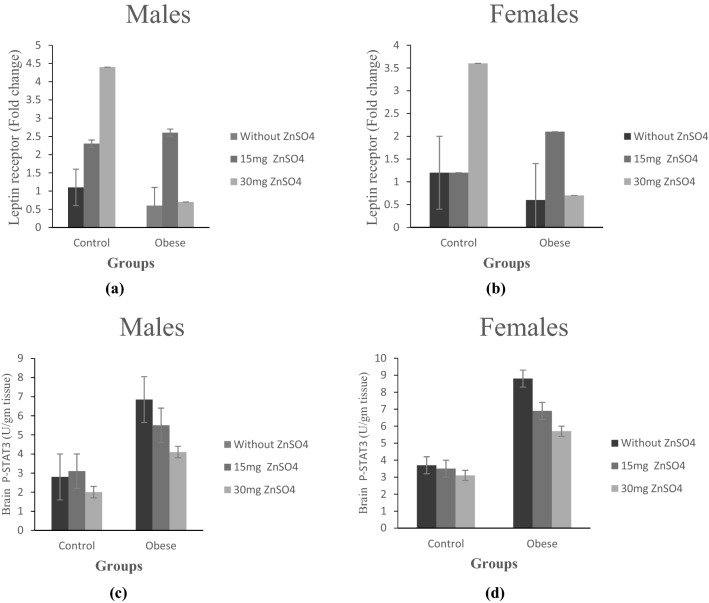
(**a**,**b**) Fold change in leptin receptor expression in brain tissue in control and obese groups; without ZnSO_4_, 15 mg ZnSO_4_, and 30 mg ZnSO_4_ in male and female rats, respectively. (**c**,**d**) Brain tissues levels of p-STAT3 (U/gm tissue) in control and obese groups; without ZnSO_4_, 15 mg ZnSO_4_, and 30 mg ZnSO_4_; in male and female rats, respectively. Results are expressed as (mean ± S.D).

Our results indicated an insignificant downregulation of LepR gene expression and a significant elevation in p-STAT3 levels in the hippocampus of male and female obese rats as compared to their control counterparts (p ≤ 0.05), Fig. (3a–d).

Low dose of supplemented Zn significantly upregulated LepR gene expression, whereas both doses significantly decreased p-STAT3 levels in obese rats of both sexes as compared to untreated groups (p ≤ 0.05), Fig. (3a–d).

**Figure 4:**
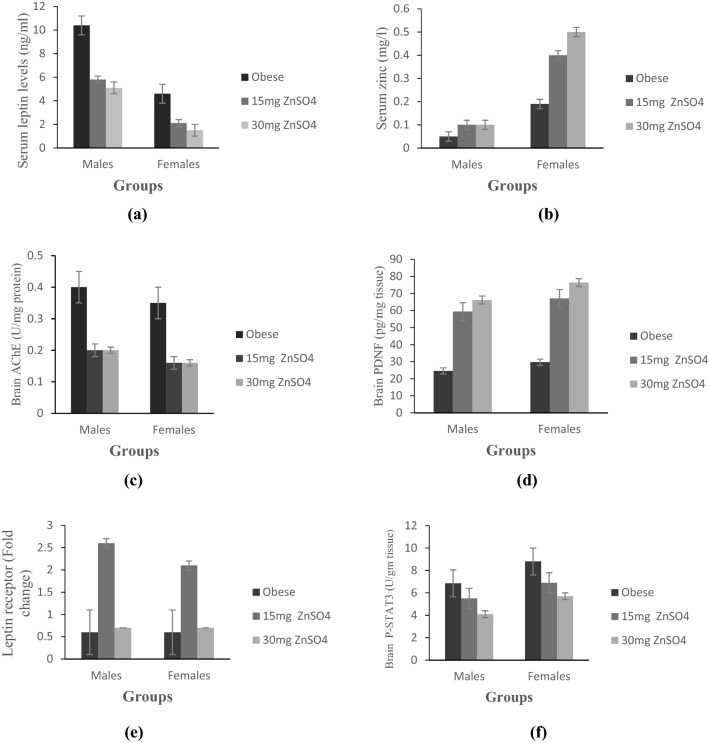
Comparison between male versus female obese rats before and after 15 mg ZnSO_4_, and 30 mg ZnSO_4_ regarding; (**a**) serum leptin levels; (**b**) serum zinc content; (**c**) brain AChE (U/mg protein); (**d**) brain PDNF (pg/mg tissue); (**e**) fold change in leptin receptor gene expression in brain tissues; (**f**) brain p-STAT3 (U/mg tissue). Results are expressed as (mean ± S.D).

Regarding sex variation, no significant difference in LepR gene expression was found between obese male and female rats, Fig. (4e). However, a significant increase in hippocampal p-STAT3 levels in obese females was detected compared to obese males (p ≤ 0.05). After treatment, p-STAT3 levels were significantly increased in obese females compared to obese males, Fig. (4f).

### Histopathological results

**Figure 5:**
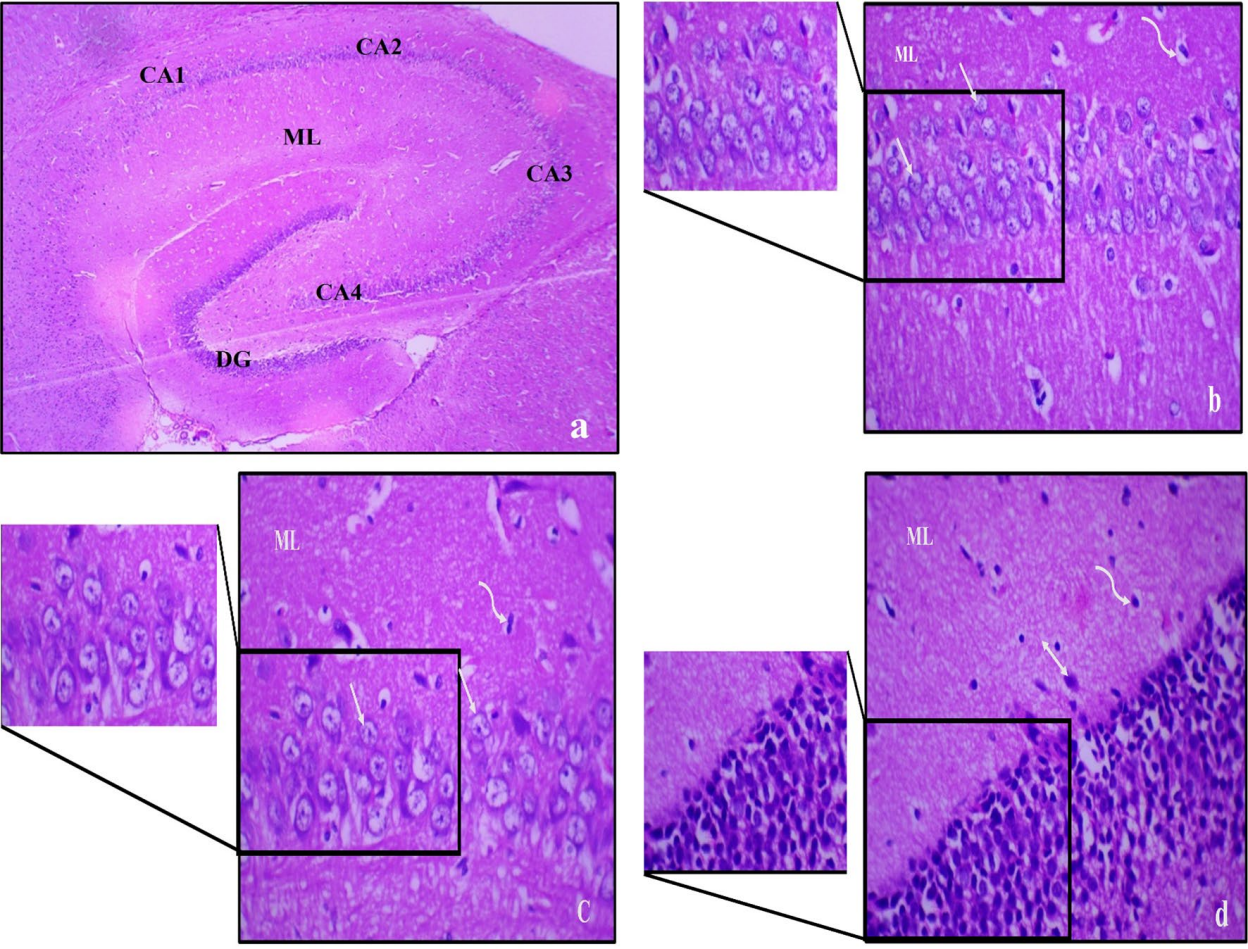
Photomicrograph of haematoxylin and eosin-stained sections from hippocampus of control male rat showing normal regions and histological structure of the hippocampus, where; (**a**) The hippocampus proper is formed of the Cornu Ammonis (CA) as CA1, CA2, CA3 & CA4 regions, and is continued as subiculum (S). Dentate gyrus (DG) is seen around CA4 by its upper & lower limbs. Molecular layer (ML) is in between CA and of DG (H & E ×40). (**b**) 4–5 compact layers of small pyramidal neurons of CA1 region, most with vesicular nuclei (↑); (**c**) shows few layers of large pyramidal neurons in CA3 region, also with vesicular nuclei (↑); (**d**) shows layers of compact granular cells with dark nuclei in dentate gyrus. Molecular layer (ML) shows many glial cells (wavy arrow) as well as pyramidal cells (↕) [H & E; (**a**) ×40, (**b**–**d**) ×400].

**Figure 6:**
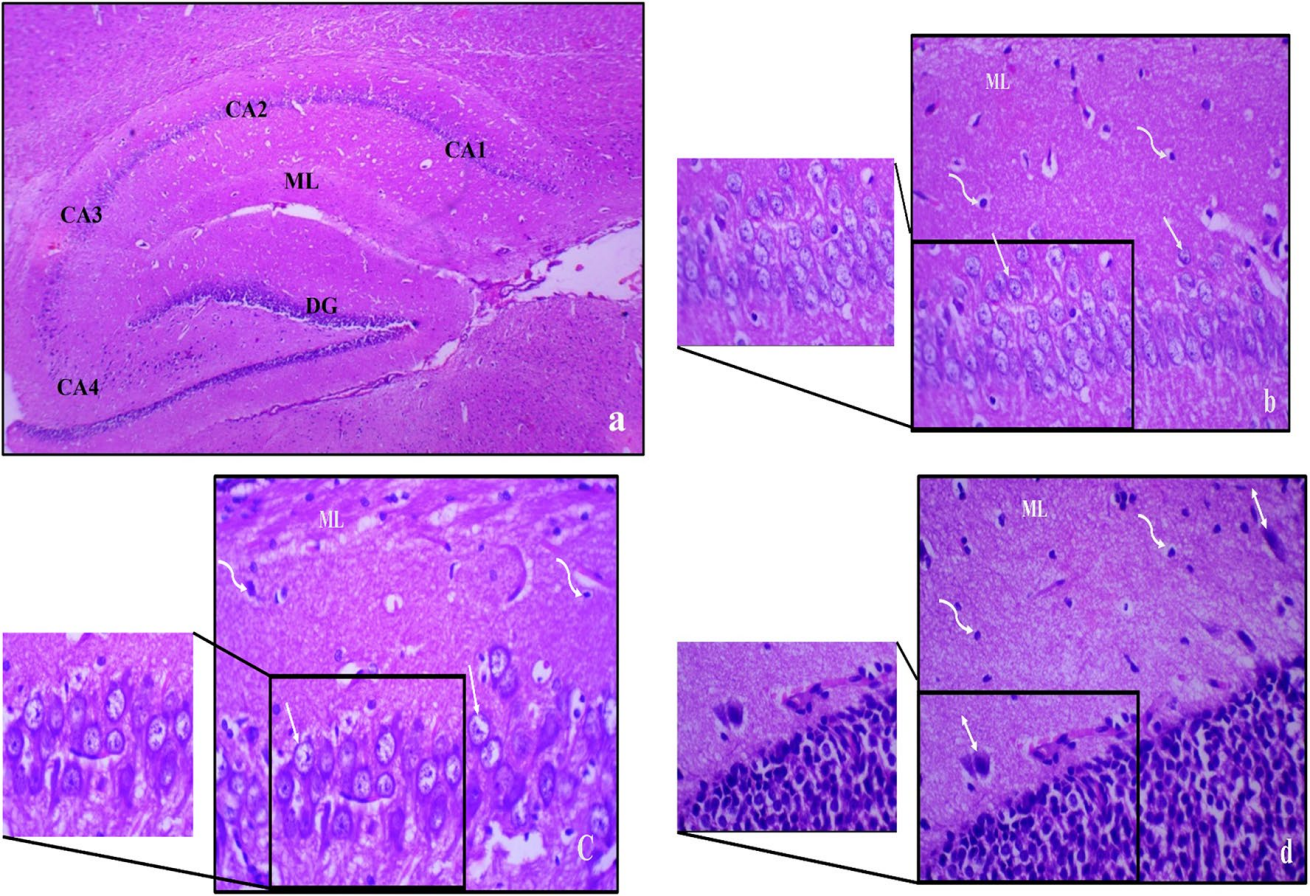
Photomicrograph of haematoxylin and eosin-stained sections from hippocampus of control female rat showing normal regions and histological structure of the hippocampus, where; (**a**) The hippocampus proper is formed of the Cornu Ammonis (CA) as CA1, CA2, CA3 & CA4 regions, and is continued as subiculum (S). Dentate gyrus (DG) is seen around CA4 by its upper & lower limbs. Molecular layer (ML) is in between CA and of DG (H & E ×40). (**b**) 4–5 compact layers of small pyramidal cells of CA1 region, most with vesicular nuclei (↑); (**c**) shows few layers of large pyramidal cells in CA3 region, also with vesicular nuclei (↑); (**d**) shows layers of compact granular cells with dark nuclei in dentate gyrus. Molecular layer (ML) shows many glial cells (wavy arrow) as well as pyramidal cells (↕) [H & E; (**a**) ×40, (**b**–**d**) ×400].

Haematoxylin and eosin (H&E)-stained sections from control male and female rats showed normal histological structure of the hippocampus, Figs. (5, 6). The main regions of the hippocampus are hippocampus proper, dentate gyrus (DG) and Subiculum (S). The molecular layer is located in between the Cornu Ammonis (CA) and characterized by a population of scattered glial and neuron cells.

**Figure 7:**
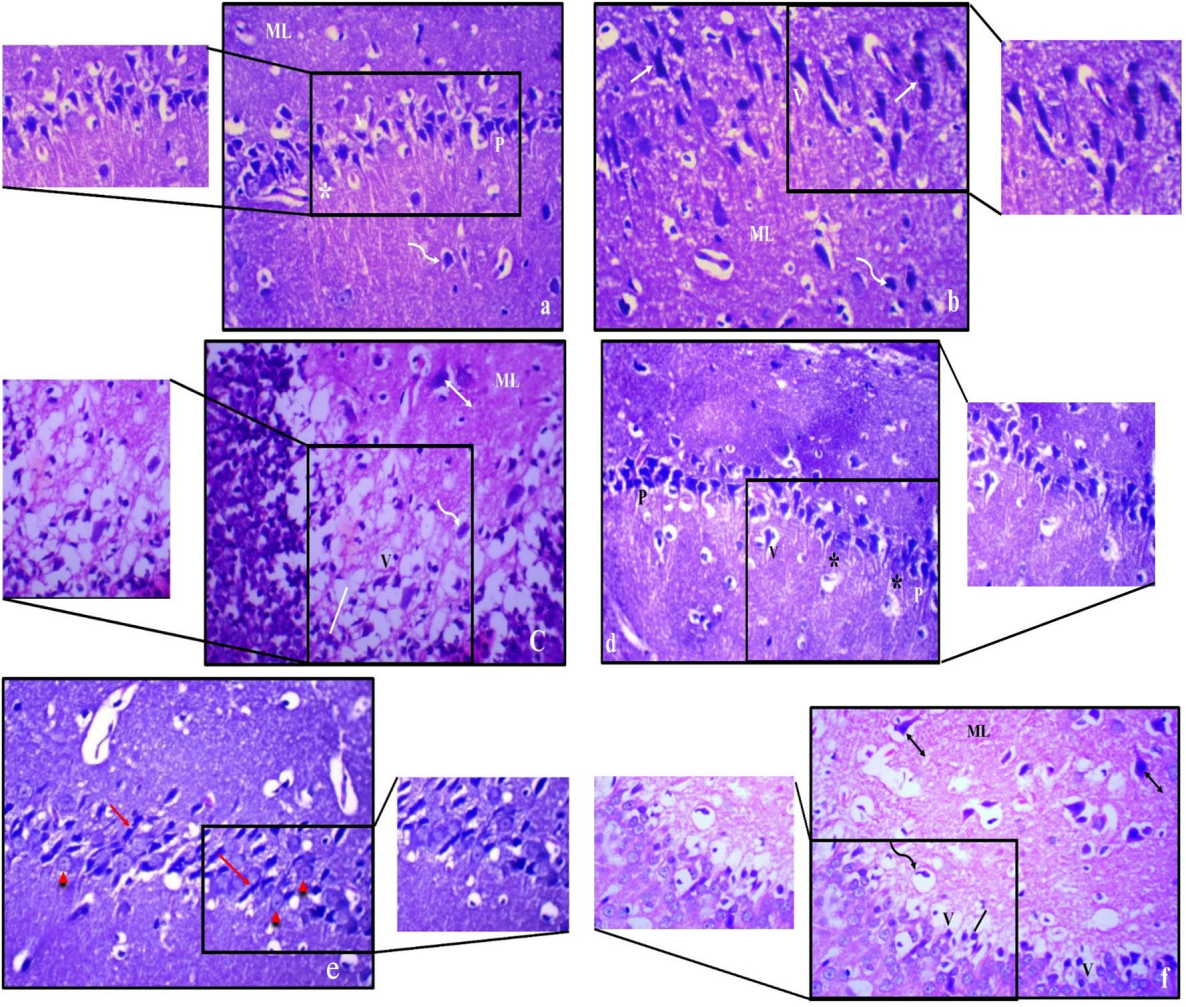
Photomicrograph of haematoxylin and eosin-stained sections from hippocampus of obese male rat (**a**–**c**) and obese female rat (**d**–**f**), where; (**a**) showing areas of small pyramidal neurons loss; some have pyknotic (P) and others have karyorrhectic (_*_) nuclei with vacuolation (V); (**b**) disorganization and extensive damage with apoptosis of most large pyramidal neurons (↑) with vacuolation (V); (**c**) granular cell layers (|) showed cell loss with marked vacuolations. Molecular layer (ML) shows marked enlargement of pyramidal (↕) and glial cells (wavy arrow); (**d**) showing areas of small pyramidal cells loss; some have pyknotic (P) and others have karyorrhectic (_*_) nuclei with vacuolation (V); (**e**) disorganization and extensive damage with apoptosis of many large pyramidal cells (↑) with few normal cells (right pointing triangle); (**f**) granular cell layers (|) showed cell loss with less vacuolation compared to males. Molecular layer (ML) shows marked enlargement of pyramidal (↕) and glial cells (wavy arrow) (H & E, ×400).

The hippocampus of male and female obese rats showed reduction of small pyramidal neurons; most were with pyknotic and karyorrhectic nuclei. Large pyramidal neurons exhibited extensive damage and disorganization; while few neurons appeared normally in female sections. The granular layer showed cell depletion with noticeable vacuolation. The molecular layer showed enlarged neurons and glial cells (Fig. 7).

**Figure 8:**
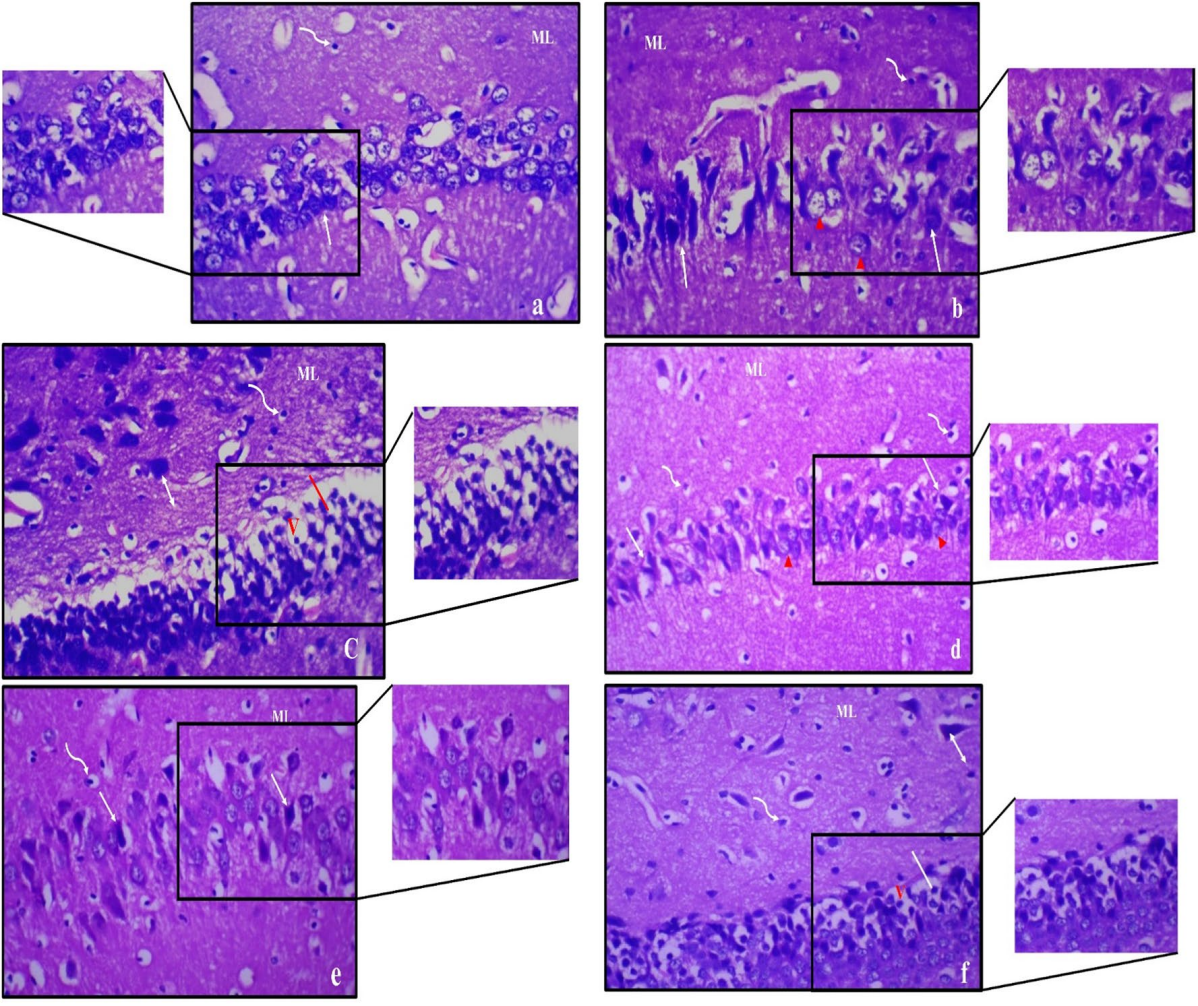
Photomicrograph of haematoxylin and eosin-stained sections from hippocampus of obese male rat (**a**–**c**) and obese female rats (**d**–**f**) treated with 15 mg of zinc showing partial improvement, where; (**a**) preservation of small pyramidal neurons with many apoptotic cells (↑); (**b**) marked apoptosis of large pyramidal neurons (↑) with few normal cells (right pointing triangle) and (**c**) decreased population of granular cells (|) with marked disorganization and vacuolation (V). Molecular layer (ML) mostly shows marked enlargement of neurons (↕) and many glial cells (wavy arrow); (**d**) preservation of small pyramidal cells (right pointing triangle) with many apoptotic cells (↑); (**e**) marked apoptosis of some large pyramidal cells (↑) and (f) **d**ecreased population of granular cells (|) with marked disorganization with less vacuolation (V). Molecular layer (ML) mostly shows marked enlargement of many neurons (↕) and many glial cells (wavy arrow) (H & E, ×400).

**Figure 9:**
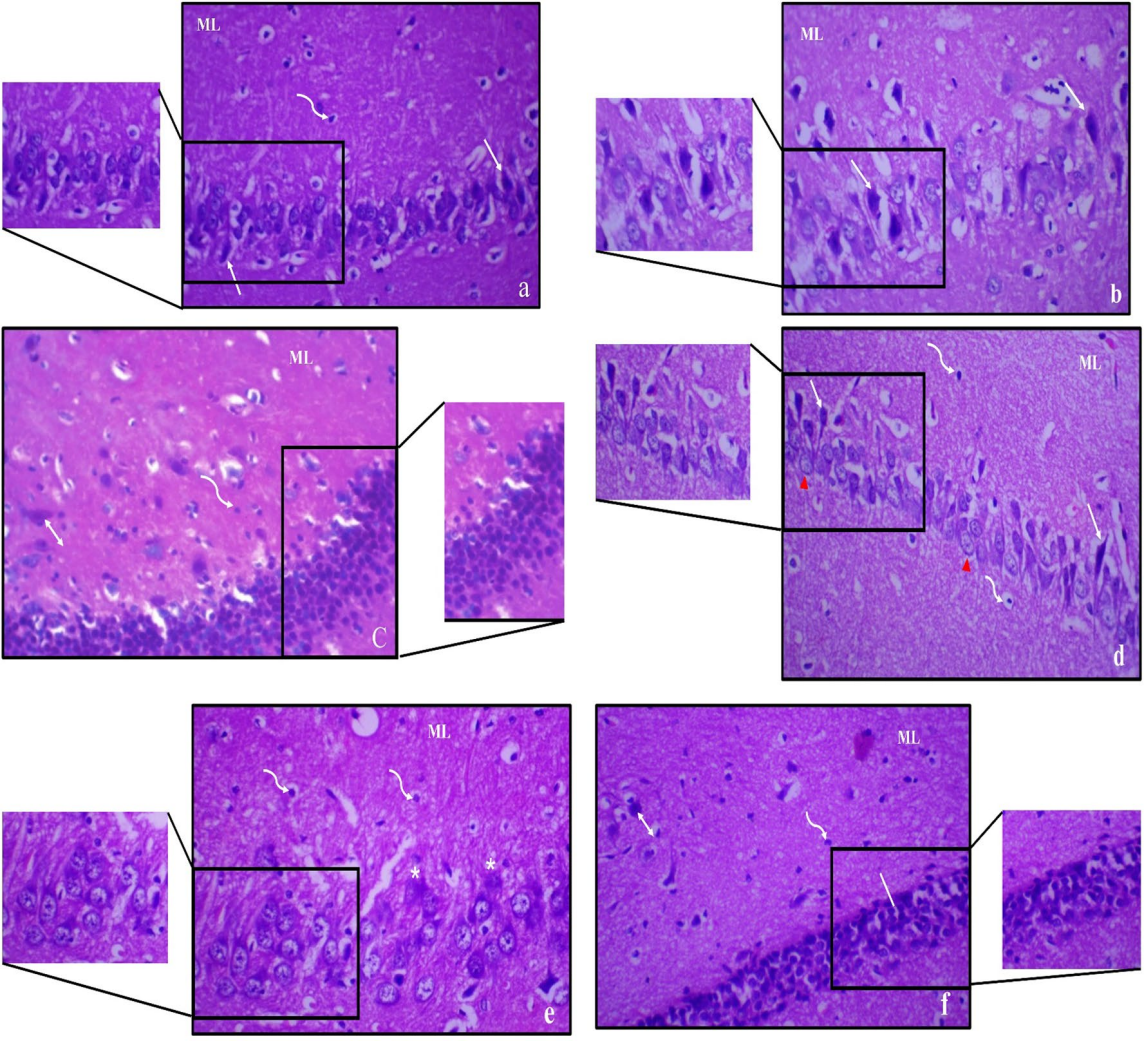
Photomicrograph of haematoxylin and eosin-stained sections from hippocampus of obese male rat (**a**–**c**) and obese female rat (**d**–**f**) treated with 30 mg of zinc showing dramatic improvement, where: (**a**) preservation of small pyramidal neurons with few apoptotic cells (↑); (**b**) some large pyramidal neurons with pyknotic nuclei (↑) and (**c**) preservation of granular cells with mild disorganization and vacuolation (V). Molecular layer (ML) mostly shows some enlarged neurons (↕) and many glial cells (wavy arrow); (**d**) preservation of small pyramidal cells (right pointing triangle) with few apoptotic cells (↑); (**e**) large pyramidal cells show few karyorrhectic nuclei (_*_) and (**f**) preservation of granular cells (|) and the molecular layer (ML) mostly shows few enlarged neurons (↕) and many glial cells (wavy arrow) (H & E, ×400).

Treatment with 15 mg of Zn showed partial improvement in the hippocampus histologic structure as preservation of small pyramidal neuron with numerous pyknotic nuclei was noticed, Fig. (8). However, large pyramidal neurons in male rats exhibited marked apoptosis when compared to female rats. Treatment with 30 mg of Zn showed significant recovery of both small and large pyramidal neurons and preservation of granular cells and molecular layer of female compared to male rats, Fig. (9).

## Discussion

Dietary Zn deficiency is increasingly associated with metabolic diseases including obesity^22–24^ and cognitive impairments^6^. Hence, our study investigated the potential effect of Zn supplementation on the cognitive biomarkers and leptin signaling pathway in high fat diet (HFD)-induced obesity rat model.

In advance, the present results revealed that HFD led to a significant increase in the body weight and induced hyperglycemia, dyslipidemia and hyperleptinemia in male as well as female rats, without altering Zn levels. Normal Zn levels might be attributed to its dynamic nature and the different factors regulating plasma or serum Zn levels such as stress, catabolism, hormones and food intake^25^.

Following low- and high-dose Zn intake (15 and 30 mg/kg ZnSO_4_⋅7H_2_O) for 4 weeks, the body weight and the serum levels of TG, glucose and leptin were significantly decreased in obese rats of both sexes compared to untreated ones. Similarly, Thoen et al.^12^ demonstrated that low-dose Zn supplementation (6 mg/kg) lowered adiposity, enhanced insulin sensitivity, and reduced leptin levels in obese animals. Qi et al.^26^ also reported that high-dose Zn supplementation (90 mg/Kg) decreased lipid accumulation, hyperglycemia and hepatic glucose production caused by HFD feeding, implying that Zn is beneficial to obese people.

It has been proposed that Zn is essential for controlling appetite and preventing obesity through its interaction with leptin hormone^27^. In obese mice, Liu et al.^9^ found a negative association between Zn and leptin levels, indicating that Zn deficiency increases leptin production and aggravates adipose tissue inflammation. Zn also acts as an insulin-mimetic through stimulating phosphorylation of one or more insulin signaling pathway components^28,29^. Moreover, Zn intake has been shown to reduce hyperglycemia, insulin resistance, beta-cell dysfunction, dyslipidemia and progression to diabetes^30^.

Recently, Zn has been reported to play a key role in the brain synaptic activity, neuronal plasticity, functions including learning and memory^6^. Previously, Zn deficient mice showed cognitive deficits, whereas sufficient Zn consumption in patients with Alzheimer’s disease decreased the rates of cognitive decline^31^. In the current research, HFD feeding led to a significant increase in acetylcholinesterase (AChE) levels and a significant reduction in brain-derived neurotrophic factor (BDNF) levels in hippocampus of male and female rats, which were reversed by both doses of Zn treatment. These findings indicate that Zn supplementation might be beneficial in improving the cognitive impairments associated with obesity.

In agreement with the present results, de Oliveira et al.^32^ showed that low-dose Zn treatment for 4 weeks reduced neuroinflammation and memory deficit caused by obesity. Nam et al.^20^ reported that administration of low Zn increased hippocampal neurogenic and synaptic marker proteins by decreasing lipid peroxidation and upregulating BDNF expression in obese mice. Moreover, higher dietary Zn intake (30 p.p.m.) was found to prevent cognitive impairments in transgenic mouse model of Alzheimer’s disease through upregulating BDNF and alleviating amyloid beta and mitochondrial dysfunction^33^.

Zn is a crucial factor in regulating the expression of hippocampal BDNF gene^34^. Zn chelators have been found to impede the processing of pro-BDNF, thereby decreasing active BDNF, impairing synapse formation and cognitive performance^35^. Zn treatment prevented neurotoxicity in rats through downregulation of AChE activity in cerebrum and AChE inhibitors can regulate Zn homeostasis, leading to an improvement of cognitive function^36,37^.

In previous studies, researchers studied the effect of excessive Zn and showed that 60 ppm of Zn aggravated obesity-induced deficits in hippocampal synaptic plasticity and neurogenesis by decreasing synaptic markers and BDNF levels^20^. So, further investigations are still required to determine the optimal dose and duration of supplemented Zn.

The adipokine leptin has been identified as an important regulator of hippocampal synaptic plasticity and memory^4^. Leptin receptors (LepRs) are expressed in the hippocampus's CA1, CA3, and dentate gyrus regions. They can activate JAK2, which phosphorylates STAT3, increasing its ability to increase transcription of genes controlling cognitive functions^38^.

Our findings revealed a significant downregulation of LepR expression in HFD-fed rats, but this downregulation was not significant. Meanwhile, low and high doses of Zn supplementation increased LepR expression in obese rats, suggesting that both doses of Zn treatment can modulate LepR expression in hippocampus and effectively ameliorate cognitive impairments. Surprisingly, we demonstrated that HFD-induced obesity resulted in a significant increase in hippocampal p-STAT3 levels, which was reversed by Zn treatment. Since p-STAT3 is not unique to leptin signaling, it is possible that other signaling cascades cause STAT3 to be phosphorylated. However, this requires further investigations.

In recent years, many studies have investigated the gender differences in cognitive decline. Most researchers suggested that metabolically unhealthy women are at a greater risk for dementia than men^39,40^. Others found that obesity, hypertension, and high adiposity increased the risk of cognitive impairment in men but not in women^41^. In our study, the male rats were more susceptible than females to weight gain and metabolic alterations, including increased total lipids, total cholesterol and leptin levels. In addition, we found that HFD feeding led to a greater reduction in BDNF levels and elevation in AChE activity in the hippocampus of male rats compared to females, suggesting that obese males are at a greater risk for cognitive decline. This gender disparity could be explained by estrogen's effects, which shield female rats from the HFD impact and cause less change in learning and hippocampal synaptic plasticity^42^.

Moreover, our findings revealed that female obese rats were more responsive to Zn treatment than males. This was manifested by the prominent increase in BDNF levels and decrease in AChE, total lipids, TC and leptin levels in obese female rats received either low or high doses of Zn.

The present results were confirmed by histopathological investigation of the hippocampal tissue. In obese male rats, extensive damage of most pyramidal neurons, cell loss of granular layers with vacuolations, and marked enlargement of pyramidal and glial cells in molecular layer were observed, indicating that deterioration of hippocampus in obese males was more severe than females. Zn treatment in obese male rats showed partial improvement in hippocampus histological structure, while Zn treatment in obese female rats resulted in a significant recovery of small and large pyramidal neurons concomitant with preservation of granular cells and the molecular layers, indicating that females were responsive to Zn treatment than males.

In conclusion, the present study provides the first evidence that low and high doses of supplemented Zn attenuate obesity-related central leptin resistance and cognitive decline. Additionally, female obese rats were more responsive to Zn treatment than male obese rats. We would recommend building up on the basis of this study findings and further exploring the ameliorative impact of Zn treatment on cognitive impairment associated with obesity. Furthermore, using silencing approach model to highlight the role of leptin signaling pathway as a mediator should be considered in future studies.

## Data Availability

The datasets used and/or analyzed are available from the corresponding author on reasonable request.
